# VMMC clients' perception of increased risk of HIV infection, circumcision preferred choice of method, providers' socio-demographics and mode of service delivery

**DOI:** 10.4314/ahs.v20i4.8

**Published:** 2020-12

**Authors:** Barbara M Nanteza, Ronald H Gray, David Serwadda, C Kennedy, Fredrick Makumbi

**Affiliations:** 1 Makerere University College of Health Sciences, School of Public Health, Department of Epidemiology & Bio statistics; 2 Johns Hopkins University, Bloomberg School of Public Health, Department of Epidemiology; 3 Makerere University College of Health Sciences, School of Public Health, Department of Disease Control and Environment Health; 4 Johns Hopkins University, Bloomberg School of Public Health, Department of International Health

**Keywords:** Male circumcision, challenges, HIV prevention

## Abstract

**Introduction:**

Voluntary medical male circumcision (VMMC) is a scientifically proven HIV prevention intervention. Uganda, like many countries has been implementing VMMC for over 10 years but uptake is still low especially in northern Uganda. To attain 80% needed for public health impact, scale-up was recommended with many innovations implemented with sub-optimal results. This study therefore wanted to find out some of the correlates of VMMC uptake in Gulu district, northern Uganda.

**Methods:**

Two studies were conducted separately but data was analyzed for this study. For the quantitative study, proportions and frequencies were used to measure perception of increased risk of HIV infection using age, gender, occupation, marital and circumcision status. Qualitative study provided data from FGDs, IDIs and KIIs were first transcribed in Acholi and then translated in English. Transcripts were uploaded in MAXDQA software for data management. A code book for emerging themes was developed.

**Results:**

A total of 548 respondents were interviewed for the quantitative study, where two thirds (66%) of the participants perceived themselves to be at increased risk of HIV infection. For the qualitative study, 149 participants from 19 FGDs, 11 KIIs and 9 IDIs were interviewed. Data were analyzed thematically using both inductive and deductive approaches. Devices were preferred to conventional surgery while mobile services were preferred to static services. However, there were divergent views regarding circumcision service providers' socio-demographics and these were influenced mainly by age, level of education and location.

**Conclusion:**

People in Northern Uganda perceived themselves to be at an increased risk of HIV infection. They preferred devices to conventional surgery, mobile services to static services but had varying views about the socio-demographics of the service providers.

## Introduction and Background

Globally close to 38 million people are living with HIV/AIDS, 70% of whom are found in sub-Saharan Africa. HIV/AIDS has prevailed for more than 40 years with the first case in Uganda discovered over three decades ago in Rakai district, Central Uganda. In the 1980/1990s, the national HIV/ AIDS prevalence peaked at 18% but had declined to 6.2% by 2017. Many HIV prevention interventions like abstinence, being faithful and condom use (ABC), elimination of mother to child transmission (e-MTCT), Anti-retroviral therapy (ART), Pre-exposure prophylaxis (PrEP), Post exposure prophylaxis (PEP), screening and treatment of sexually transmitted infections (STIs) and voluntary medical male circumcision (VMMC) have been implemented throughout the country. However, HIV prevalence in mid north Uganda is still higher (7.2%) than the national prevalence which is at 6.2% 1. Even though the national VMMC coverage is at 42.2% and varies among regions, VMMC coverage in mid north is still very low at 13.1%. In order to improve uptake of VMMC in this region, correlates of VMMC uptake in this region should be known and used for policy formulation, re- viewing national VMMC strategic documents that guide VMMC implementation and more importantly used in development of mobilization messages. In 2007, WHO and UNAIDS recommended VMMC for countries with high HIV prevalence but low VMMC coverage^2^. This was after the three clinical trials that provided evidence that circumcision can reduce HIV acquisition of a male from an infected female by up to 60%^3^,^4^,^5^. For any country to have a public health benefit from circumcision, 80% of its eligible males should be circumcised. This means that 7 million Ugandan males have to be circumcised by end of 2020 although only 4.9 million males have been circumcised as of October 2019. This target has to be allocated to all the ten regions (central 1 & 2, Kampala, East Central, Mid-Eastern, Mid-west, Mid- North, North East, West Nile and South West) depending on the coverage needed to attain the 80% in that region. VMMC is being funded by PEPFAR in all the 15 East and Southern African priority countries including Uganda. Although many countries started VMMC implementation in 2008 after the recommendation by WHO and UNAIDS, Uganda started in 2010 after the development of VMMC policies and guidelines.

These regional variations in VMMC coverage correlate with HIV prevalence and it's unfortunate that the regions with high HIV prevalence also have low VMMC coverage^6^. Mid-Eastern region has the highest VMMC coverage in the country and its HIV prevalence is below the national average.

Despite implementation of many interventions and strategies, Uganda has not yet attained HIV epidemic control. Among the behavioral interventions that have progressively been implemented to address the HIV epidemic are; Abstinence; Be-faithful and Condoms (ABC) strategy; Elimination of Mother-to-Child Transmission (e-MTCT); Antiretroviral Therapy (ART); Post Exposure Prophylaxis (PEP); Pre-exposure Prophylaxis (Pr-EP), and Sexually Transmitted Infections (STIs) screening and treatment. HIV testing services (HTS) are not treatment but a precursor to adopting to HIV prevention methods. ABCs have not been effective in reducing the spread of HIV infection, their cumulative effect has diminished spread in some instances and modified behavior in other instances^7^,^8^,^9^ Voluntary Medical Male Circumcision is by far the most efficient and effective biomedical intervention in reducing the HIV epidemic since it a one-off procedure^10^.

For the past 6 years, Uganda has been scaling up VMMC services in the entire country. However, VMMC uptake is still low especially in northern Uganda. This study therefore aimed to identify the correlates of VMMC uptake in Northern Uganda such that the implementing partners, district officials, major stakeholders and policy makers could use the evidence to ensure that VMMC uptake increases in this region.

## Methods

### Study design and setting

Two separate studies were conducted in Gulu district in four static sites that were offering VMMC services. Gulu district is found in mid-north of Uganda, a region scarred from a twenty-year civil war that fueled the HIV epidemic and left many internally displaced people. The quantitative and qualitative studies were conducted in December 2015 and September 2016 respectively. Both were conducted in the same catchment areas within a 5kilometer radius of the four public health facilities that are the only ones that provide static VMMC services in the district. The facilities are; Lacor Hospital, Gulu Regional Referral Hospital, Awach Health Center IV, and Lalogi Health Center IV.

Quantitative data was got from men aged 18 years or older and women who were married and aged 18 year or more but had to have stayed for more than 6 months in Gulu district. A semi-structured questionnaire which was developed for this study was pre-tested and piloted at a non-study site. This was used to collect cross-sectional data on socio-demographic characteristics, circumcision status, knowledge on VMMC and perception of being at increased risk of HIV infection. The questions were administered in a respondent's preferred language. The questions analyzed for this manuscript were about perception of increased risk of HIV infection though the other questions were mainly about knowledge of VMMC messages and has already been published^11^. Systematic sampling was used to select households using lists that had been used earlier during a mosquito net distribution campaign. Eligible residents were ranked by age in descending order, and potential participants were randomly selected using a KISH grid. These were then contacted for a possible interview, with two additional attempts for respondents who were not initially reached. Qualitative data were collected from 149 individuals aged 10 to 69 years who participated in 19 focus group discussions (FGDs), 11 key informant interviews (KIIs), 9 in-depth interviews (IDIs) until saturation. Each FGD consisted of 6–8 individuals of similar age, gender, and marital status (Table 1). Circumcision status was not considered because its coverage is very low in this area and could possibly lead to stigma and discrimination. IDIs were conducted with men and women who held strong opinions for and against circumcision, and these were identified by village health teams (VHTs).

**Table 1:** Summary of FGDs, IDIs and KIIs

Focus Group Discussions (FGDs)	Number conducted 129 participants total
Children boys (10–12 years)	1
Adolescent boys (13–19 years)	3
Unmarried men (20–25, 26–34, 35+ years)	3 (one per subgroup)
Married men (20–25, 26–34 years)	3 (one per subgroup)
Married men (35+ years)	3
Unmarried women (18–24, 25–34, 35+ years)	3 (one per subgroup)
Unmarried women (18–24, 25–34, 35+ years)	3 (one per subgroup)
**Key Informant Interviews (KIIs)**	**11 participants total**
Cultural leaders	5
Religious leaders	6
**In-depth interviews (IDIs)**	**9 participants total**
Pro-circumcision	3
Anti-circumcision	6

Cultural leaders from Acholi cultural institution and religious leaders from the main religions (Catholic, Anglican, Pentecostal, Muslim, Seventh Day Adventist, and traditional) were selected for KIIs. Study team members were trained persons recruited from northern Uganda (Acholi region) and were fluent in both Acholi and English.

FGD, KII, and IDI guides had questions asking about the preferred circumcision method, providers' demographics and mode of service delivery. Participants were informed of the purpose of the study, extent of their involvement, and their rights before obtaining written informed consent. Interviews were conducted in participant's preferred language and were audio-recorded. They lasted between 60–90 minutes for FGDs and 45 minutes for IDIs and KIIs. Participants received refreshments and 7,000 Ugandan shillings (∼US $2) for their time. At the end of each working day, the study team would meet and discuss findings. Already some findings from this qualitative study have already been published (12).

### Data analysis

**Quantitative data:** Frequencies and percentages were used to measure the perception of increased risk of used to describe categorical socio-demographic characteristics - HIV infection among the participants especially with of the participants, and means (SD) location, age, gender, tribe, level of education, occupation and circumcision status. Their responses to whether VMMC could reduce this risk was also analyzed. Qualitative data from FGDs, IDIs, and KIIs were first transcribed in Acholi then translated into English. Transcripts were uploaded in MAXDQA software for data management. The first author developed a codebook based on initial team discussions and themes. The resulting codes with coded data were discussed with coauthors, summarized and presented in the results below.

### Ethical considerations

The Makerere University School of Public Health Higher Degrees Research and Ethics Committee (HDREC) and the Uganda National Council for Science and Technology (UNCST) approved the research protocol (# TR 2015002451). Permission for data collection was obtained from the district health office, the residential district commissioner, and local community leaders.

## Results

The results are in two sections; quantitative for perception of increased risk and if VMMC could reduce the risk of increased HIV/AIDS infection while qualitative was used to determine preferred circumcision method, providers' socio-demographics and mode of service delivery.

### Quantitative results

A total of 548 respondents were interviewed with a response rate of 92.8%. Their socio-demographic characteristics are in the table below.

**Table 2.0:** Study participant social and demographic characteristics

		Communities		
Characteristics	All combined	Lalogi	Awach	Municipality
	N (%)	N (%)	N (%)	N (%)
**Age (Years)**	**548(100)**	**131(23.9)**	**120(21.9)**	**297(54.2)**
15–24	244 (44.6)	63(48.1)	60(50.0)	121(40.7)
25–34	221 (40.3)	43(32.8)	39(32.5)	139(46.8)
35–44	66 (12.0)	19(14.5)	15(12.5)	32(10.8)
45+	17 (3.1)	6(4.6)	6(5.0)	5(1.7)
				
**Sex**	**547 (100)**	**131(23.9)**	**119(21.8)**	**297(54.3)**
Male	472 (86.3)	120(91.6)	92(77.3)	260(87.5)
Female	75(13.7)	11(8.4)	27(22.7)	37(12.5)
**Marital Status**	**547(100)**	**131(23.9)**	**119(21.8)**	**297(54.3)**
Married	343(62.7)	49(37.4)	48(40.3)	84(28.4)
Not Married	204(37.3)	82(62.6)	71(59.7)	213(71.6)
				
**Tribe**	**548(100)**	**132(24.1)**	**119(21.7)**	**297(54.2)**
Acholi	471(86.0)	105(80.0)	115(96.6)	251(84.5)
Langi	51(9.3)	25(19.0)	0(0.0)	26(8.8)
Others	26(4.7)	2	4	20(6.7)
				
**Religion**	**546(100)**	**132(24.2)**	**118(21.6)**	**296(54.2)**
Catholic	368(67.4)	82(62.1)	89(75.4)	197(66.6)
Anglican	119(21.8)	31(23.5)	20(17.0)	68(23.0)
Evangelicals/SDA	47(8.6)	15(11.4)	8(6.8)	24(8.1)
Muslims	12(2.2)	4(3.0)	1(0.8)	7(2.4)
				
**Education**	**542(100)**	**130(24.6)**	**117(21.6)**	**295(53.8)**
None	31(5.7)	9(6.9)	12(10.3)	10(3.4)
Primary	137(25.3)	50(38.5)	47(40.2)	40(13.6)
Secondary	242(44.7)	60(46.2)	50(42.7)	132(44.6)
Tertiary	132(24.4)	11(8.5)	8(6.8)	113(38.3)
				
**Occupation**	**530(100)**	**123(23.2)**	**115(21.7)**	**292(55.1)**
Peasant	32(6.0)	13(10.6)	16(14.0)	3(1.0)
Farmer	100(18.9)	57(46.3)	35(30.4)	8(2.7)
Casual Laborer	32(6.0)	10(8.1)	3(2.6)	19(6.5)
Market Vendor	15(2.8)	0(0.0)	3(2.6)	12(4.1)
Shop Attendant	23(4.3)	4(3.3)	2(1.7)	17(5.8)
Teacher	32(6.0)	5(4.1)	5(4.4)	22(7.5)
Business Person	71(13.4)	9(7.3)	12(10.4)	50(17.1)
Health Care Worker	33(6.2)	7(5.7)	5(4.4)	21(7.2)
Civil Servant	17(3.2)	0(0.0)	1(0.9)	16(5.5)
Student	74(14.0)	15(12.2)	18(15.7)	41(14.0)
Boda boda Rider	36(6.8)	2(1.6)	5(4.4)	29(9.9)
Unemployed	59(11.1)	1(0.8)	6(5.2)	52(17.8)
Others	6(1.1)	0(0.0)	4(3.5)	2(0.7)
				
**Circumcision Status**	**528(100)**	**125(23.7)**	**115(21.8)**	**288(54.5)**
Circumcised	157(29.7)	31(24.8)	31(27.0)	95(33.0)
Not Circumcised	371(70.3)	94(75.2)	84 (73.0)	193 (67.0)

Table 1:0 shows study participant social and demographic characteristics. Overall, nearly half (446%) of the respondents were young people aged 15–24 years. This was consistent in communities of Lalogi (48.1%) and Awach (50.0%) that were both rural, but not in Gulu municipality where majority (46.8%) were aged 25–34 years. Just over a quarter (22.7%) of respondents in Awach were married women compared to only 8.4% in Lalogi and 12.5% in Gulu municipality. Unlike in the Municipality, almost everyone in Lalogi and Awach was native. Universally (97.8%) all respondents were not Muslims primarily Catholics (67.4%); this is consistent throughout the three study communities. Slightly above two thirds (69.1%) of the respondents had post primary education and this was highest (82.3%) in Gulu and lowest 49.5%) in Awach. Farming was the commonest occupation but also unemployment was high (17.8%). Overall 29.7% reported either being circumcised or their partners, highest (33.0%) in Gulu and lowest (24.8%) Lalogi.

### Perception to be at increased risk of HIV infection with location

The municipality had majority of those who said that they did not know their status (56.6%) and those that were very likely to be exposed. Trends changed when it came to those who said that they felt they were exposed with almost all communities being at the same level.

### Perception to be at increased risk of HIV infection with Age

Those who said they did not know if they were at risk of HIV infection, half (50.0%) were aged 15–24 years with none aged between 45 and 57 years. Those who perceived themselves as exposed were almost the same across all age groups

### Perception to be at increased risk of HIV infection with Gender

Nine out ten (89.3%) people interviewed were men. Those who did not know whether they were at risk of HIV infection, one out of five (20.8%) were women. Three quarters (75.0%) of the women perceived themselves as exposed while 81.5% of the men thought the same.

### Perception to be at increased risk of HIV infection with Marital Status

Slightly more than half (55.1%) were married although 10.5% of them did not know if they were exposed. Four fifths (80.6%) of the married were exposed and 81.1% of the unmarried were also exposed.

### Perception of increased risk of HIV infection with Occupation

Farmers (29.2%) and students (12.5%) perceived themselves more to be at risk of HIV infection but unemployment was a factor.

Those who thought VMMC could reduce the risk of HIV infection

Nine out of ten (91.5%) of the participants said they knew VMMC could reduce the risk of HIV infection, with less than 5% saying no or did not know. Universally all respondents said that they knew that VMMC can reduce the risk with no difference between the circumcised and non-circumcised.

### Qualitative Results

Findings are presented according to the responses to the clients' preferred circumcision method, providers' demographics and mode of service delivery.

### Clients' preferred circumcision method

Voluntary Medical male circumcision has two methods. The conventional method where surgical (re-usable or disposable) instruments with injectable anaesthesia are used to remove the foreskin while the device method is where a ring (device) with a cream or injectable anaesthesia is used.

In Uganda both methods are used but the device method is limited to few regions. Although the conventional method is the most commonly used method in this setting, participants were first sensitized and health educated on all the three circumcision methods before the interviews (FGDs, IDIs and KIIs). The three methods included conventional, Prepex and Shangring. In brief, conventional method is where we inject local anesthesia, excise the prepuce, appose and stich back the outer and the inner skin, dress the wound and instruct the client to come back after 48 hours to remove the bandage. Shangring, is a device where we have to first measure the size of the penis to know the right ring size to use. There after injectable or a cream for local anesthesia can be used before marking the area where the rings (inner and outer) will be placed. The prepuce is then cut off immediately after rings have been placed. The client is instructed to return on the 7th day for rings removal where service provider demonstrates how to apply the bandages and are given bandages to apply while at home for the next 6 days. Prepex, is almost like Shangring but with Prepex we only use the cream for local anesthesia and do not cut off the prepuce on the first day. Instead the prepuce is cut off after 7 days from rings' placement and the wound heals by secondary intention. In all methods, clients are requested to return whenever they have a problem though healing is expected after 6 weeks. During all this time, clients are instructed to abstain from sex.

**Conventional:** During the discussions, the views on this method seemed to be very divergent. Some participants seemed to like it while others did not like it. Those who liked this method said that it was quick and they did not have to come back. However, those who did not like it was mostly due to fear of pain associated with the injection that is given at the base of the penis as local anesthesia. Others preferred the conventional method simply because it has been around for many years and felt that it was most popular. *Prepex:* Many participants really liked this method because of its benefits over the other methods. The most preferred benefit over the other two was the fact that there is no cutting off the prepuce on the first day. Prepex was considered to be good for them since a live person is not supposed to spill blood. However, some participants mentioned that they did not like the smell associated with it. They also noted that it takes longer to heal as compared to the conventional which makes them abstain longer.

**Shangring:** This circumcision method was liked because of no injection thus no pain and more importantly that everything is done on the same day. Participants who feared stiches seemed to like this method over the conventional method. Also participants like this method because the foreskin was cut on the same day thus no smell was expected while the device was in situ. With the Shangring no bleeding or infection are expected since these usually occur in the first five days after surgery and the ring will still be in situ. However, some clients feared circumcision by devices because it was left in situ for at least 5 days which caused anxiety that maybe it would “damage” or constrict the penis

### Providers' Demographics Age

Participants cared about the age of service providers. Many did not want young service providers to offer VMMC services. Some considered even this to be a taboo which had serious consequences to the young service providers to see the private parts of people who are like their parents. Old clients preferred older service providers but they young ones did not care about the age of the service provider.

**Gender:** Older (30 years and above) participants did not want VMMC services to be provided by female service providers. However, the adolescents preferred female service providers yet the middle-aged participants did not bother about the gender of the service providers. **Region:** Religion was very important to the less educated and older participants. These participants thought that Muslim VMMC service providers would convert them to Islam. However, the educated and younger participants did not mind about the religion of the service providers.

**Tribe:** More educated and young participants never minded about the tribe of the service provider. However, the older and less educated did not want Muslims to circumcise them for the same reason above.

**Cadre:** The cadre of the service provider, the reverse was true for all the above. The educated participants preferred more educated service providers with some thinking that Muslims would offer a better service because they reasoned that these would be more experienced thinking they have been conducting circumcision much longer than their counterparts. The less educated did not mind about the cadre since they considered all service providers to be highly qualified.

### Mode of Service Delivery

In this study a static site was referred to a as a health facility which integrated VMMC among the usual health services it offers to the community and ideally is the one supposed to report the numbers in DHIS2. Static should offer VMMC services on a daily basis or dedicated days. These sites would have trained VMMC service providers and would get the supplies from the donor (PEPFAR) but through the implementing partner. Ideally all static sites should be certified by MoH to ensure that they met the minimum standards before offering VMMC services.

Due to the targets set and allocated to static sites, sites decide where and when they would conduct mobile services through outreaches (not exceeding two days) or camps (exceeding more than three days). A mobile site can be another facility that does not have targets but should be within the catchment area of the static site and should not report the circumcision numbers into the national system.

**Static:** Participants who preferred static sites were those who were usually near that health facility. Distance for these clients was never a problem and would be those who did not care to be seen going to site to seek VMMC services. They were usually young and more educated clients. The safety of VMMC services was of great importance to them. Clients who did not want to be known and to be associated with VMMC wanted services to be offered at static sites. One participant told us “if convinced would, I would prefer to seek VMMC services at a static site because you can easily disguise “hide” and not be seen seeking VMMC services”.

## Discussion

In this study, authors wanted to know if the people in Gulu district perceived themselves as to be at increased risk of HIV infection. This would help the authors know how to promote uptake of VMMC services since VMMC is known to reduce acquisition of HIV infection by men from HIV infected females. This study was a sub-study of a research that was looking at the correlates of VMMC uptake in northern Uganda. Two other papers that looked at knowledge, enhancers and barriers of VMMC uptake in this region have already been published^11^,^12^. After answering that question, the study further wanted to know why the people in Gulu especially men did not seek VMMC services which were being offered at no cost both at static and mobile (out - reaches or camps) sites within their communities. Previous studies had shown factors that affected VMMC uptake especially in Sub Saharan Africa and these studies had shown culture, religion, education level, socio -status and comprehensive knowledge but none had studied the socio demographics of the VMMC service providers^13^, ^14^. For any healthy intervention to be effective, the target population must be aware of the benefits. Many studies have shown the benefits of circumcision with reduction in HIV acquisition as one of them^12^,^15^. Mid north has persistently had a higher HIV prevalence than the national prevalence since 2011. Men in such regions should be encouraged to seek VMMC services but men are known to have poor health seeking behavior^16^,^17^. The results of this study showed that many people perceived themselves to be at increased risk and they knew that VMMC could reduce this risk. This means that the people should be helped and guided on how to get circumcised because of the many benefits of circumcision.

Like in all the other priority countries that implement VMMC for HIV prevention, the conventional method is the most common method in Uganda including in northern Uganda. Unlike in the traditional circumcision where the clients have no choice, Uganda has started using devices that overcome some of the challenges of the conventional method^18^,^19^. The conventional method uses the injection to administer local anesthesia which has been known to cause fear and anxiety among many VMMC clients. Even though the injection takes less than a tenth of the procedure time, it's known to cause a lot of psychological pain to many clients and this prevents many men from seeking VMMC services^20^. However, clients who do not want to return to the facility after being circumcised, have always braved the pain of this injection. It has also been noted that healing after conventional surgery is much faster as compared to the healing time of the devices 12,18.

On the other hand, devices are seen to be the solution to the pain caused by the injection since they use a cream as local anesthesia. Some have even called devices bloodless since there is no visible blood during the procedure^21^. Circumcision performed using Prepex device was preferred to the conventional method because for those who believed in culture since there is no spilling of blood which they believed to be a curse if you are still alive. On the other hand, Shangring was liked because there are no stiches yet many clients fear stiches 22,23. Poor suturing that is done during conventional method usually causes hematomas and infection. Device circumcision has always produced good Cosmesis which was a major factor that made the people to prefer devices to conventional method^23^. Circumcisions by conventional method usually gives rugged appearance which many clients do not like. From the programme perspective, devices are preferred to conventional method because they improve return rates to almost 100%. Current return rates for the VMMC programmes are as low as 55%, a lot of resources have been allocated to improve return rates from just to ensure than men return for follow-up. The study found out that older and less educated participants were more concerned about service providers' demographics as compared to the younger and more educated clients. In other studies, it was found that the quality was the major factor that affected VMMC uptake of SMC services. HIV prevalence is usually higher in urban places as compared to rural areas, so when you want to increase uptake of VMMC services we may need know what the target group preferences. Earlier studies showed that the more educated the clients are, the more likely to care about qualified service providers. However, it must be known that in Uganda, a lot of resources have been released to ensure that all the VMMC service providers must be well trained in the provision of VMMC services. Gender and religion were the demographics of great importance. Majority of the people in northern Uganda are Catholics with some thinking the VMMC programme is trying to convert them to Islam. Some of the older participants seemed to still recall that Slave trade in their region was carried out by Muslims, an event that really traumatized them. Further still the older men, did not want to be circumcised by younger service providers and being a female even made it worse. These men did not want the young girls whom some referred to as their children to see their private parts. However, in one FGD, the young participants said that they wanted to be circumcised by young beautiful girls. All these varied views about the service providers' demographics emphasize that for VMMC uptake to increase in northern Uganda, all the clients' and their spouses' concerns should be addressed. In most rural communities, cadre of health professional is never an issue, actually to them all men are considered doctors and the women nurses^24^. In order to scale up VMMC services in Uganda, there some innovations that have been implemented. Some of the innovations are mobile services which may be outreaches (less than two days) or camps more than two days. The main objective of VMMC mobile services is to bring services near the people. Also, studies have shown that uptake of services increase if the services are brought near the people^25^, ^26^, ^27^. Most participants preferred mobile services as compared to static services as evidenced by MoH data that has shown that mobile services contribute more than three quarters (87.3%) of the national circumcisions.

Since circumcision is a one off, clients near to the static facility get depleted over time. So, if an IP wants to achieve their set targets, they should move further into the communities and this can only be done by offering mobile services. Men are known to have poor health seeking behaviors, so mobile services help solve this problem by bringing the services near to them^28^. Also, there other benefits that males who seek circumcision services. Laggards and older men preferred static sites to mobile sites. They said static sites offered privacy and confidentiality. They also felt that the quality of services was better at the static site although MoH emphasizes that the quality of VMMC services should be the same both at static and mobile sites. However, over the years almost two thirds (64.8%) of adverse events occur in mobile sites but it should be noted that almost 90% of the men are circumcised from mobile sites.

## Conclusion

People in mid Northern Uganda perceived themselves to be at increased risk for HIV infection and they knew that VMMC could reduce that risk. Although the conventional method is the most commonly used method for circumcision, majority of the people in this region preferred devices especially Prepex. Regarding the service providers' demographics, they were more concerned about their religion and gender. They preferred mainly Catholic and men service providers. Mobile services were preferred to static services even though more than three quarters of adverse events occur from mobile sites.

## Figures and Tables

**Table 3:** Showing perception of increased risk of HIV infection and how VMMC can reduce that risk

Variable	Perception of Risk for HIV infection (%)			VMMC can reduce the risk	
	Very Likely	Exposed	Do not Know	Yes	No
**Location**					
Lalogi (Peri-Urban)	29.0	83.8	12.5	91.6	9.4
Awach (Rural)	34.2	72.0	31.3	83.8	16.2
Municipality (Urban)	36.8	83.1	56.3	94.6	5.4
	p (0.2)			p(0.02)	

**Age Group (Years)**					
15–24	36.8	81.3	50.0	92.1	7.9
25–34	39.5	81.0	39.6	93.0	7.0
35–44	18.4	76.9	10.4	90.4	9.6
45–57	5.3	85.7	0.00	66.7	33.3
	p(0.7)			p(0.01)	

**Gender**					
Male	94.8	81.5	79.2	92.1	7.9
Female	5.2	7.5	20.8	86.8	13.2
	p(0.04)			p(0.4)	

**Marital Status**					
Married	57.9	80.6	54.2	90.6	9.4
Not Married	42.1	81.1	45.8	92.5	7.5
	p(0.9)			p(0.5)	

**Occupation**					
Peasant Farmer	36.8	76.3	29.2	85.2	14.8
Student	8.0	14.1	12.5	91.0	9.0
Trade/Business	21.1	17.1	18.6	95.1	4.9
Unemployed	36.8	17.0	14.6	98.0	2.0
Other	26.3	80.7	25.0	91.8	8.2
	p(0.2)			p(0.04)	

Circumcision Status					
Circumcised	7.9	84.3	7.8	94.5	5.5
Not Circumcised	8.8	79.2	12.0	90.1	9.9
	p(0.08)			p(0.9)	

**Table 4:** Themes and Supportive Quotes

Category	Theme	Quote
Preferred choice of circumcision method	Convectional	“When I decided to get circumcised I did not want to return to the facility, so after learning about all the three methods I chose conventional method.” [Male, 27 years, tertiary education, FGD] “All my life I have feared injections, how do you expect me to accept to be given an injection on the penis for an intervention that is not 100% in preventing acquisition HIV?” [Male, 37 years, Secondary education, IDI]
	Prepex	“Spilling blood when you are still alive is considered a curse, so I would prefer a bloodless circumcision.” [Male, 54 years, Primary education, KII] “I wish the government can make this available, my husband would get circumcised right away because his major fear is pain.” [Female, 29, primary education, IDI]
	Shangring	“I wish I had known about this method long ago, I would have already turned up for VMMC. Personally I really do not like injections and stiches.” [Male 38 years, Secondary education, IDI] “Taking care of a spouse after being circumcised can be challenging, but with this new method this is sorted.” [Female, 29 years, Primary education, FGD]
Providers' socio-demographics	Age	“In my culture it's not good for a big man to show your private parts to a minor more so your daughter, actually it's considered a curse to that child.” [Male, 43 years, primary education, KII] “If I am sick, I have no time to think of the age of the service provider, but for VMMC it's different because I am not sick. I do not want a young person working on me.” [Male, 33 years, primary education, FGD]
	Gender	“Nature is nature, I would not want a female more so a young one to circumcise me.” [Male, 19 years, Secondary education, FGD] “We feel safe if our men are worked on by fellow men, these men can easily fall in love with any woman.” [Female, 25 years, no education, FGD]
	Religion	“If a Muslim circumcises our children, we may think that they are converting them in Islam.” [Female, 32 years, no education, FGD] “Muslims have been circumcising for many years, I think they are the best, so government should promote them to conduct VMMC.” [Male, 62 years, primary education, IDI]
	Tribe	“Unless someone has a hidden agenda, why would someone come all the way from central just circumcise me? Government should train our own people to circumcise us” [Male, 53 years, no education, IDI] “If you manage to convince me, I do not want a Mugisu or Muslim to circumcise my sons. I just hate the way they do it and I do not want my sons to go through that experience.” [Female, 41 years, primary education, FGD]
	Cadre	“It does not matter, we know that all service providers are educated.” [Male, 41 years, primary education, FGD] “More qualified service providers are the ideal. Personally I would like a medical doctor circumcising me, that way I would feel very comfortable remember a man is only a man depending on the functions of his penis.” [Male, 56 year, primary education, IDI]
Mode of service delivery	Static	“Unlike my friends, I wanted to be circumcised so I preferred to be at the facility because it's easy to disguise unlike in a camp where everyone will know that you have gone for circumcisions” [Male, 17 years secondary education, FGD] “I feel comfortable getting circumcised at the static site because I will be assured to get post operation care from my usual place.” [Male, 37 years, no education, FGD]
	Mobile(outreach and Camps)	“I had always wanted to get circumcised but I only feared walking back home with a wound yet the distance is long. The moment I saw a camp near our home, I even missed school just to get circumcised.” [Male, 22 years, Secondary education, FGD] “I can never allow my grandchildren to be circumcised from a camp, many times the standards in this camps are below the minimum standards.” [Male, 46 years, secondary education, IDI] “My son was circumcised from the camp. Three days later he got a problem that needed attention, but we found no one at the camp site.” [Female, 34 years, primary education, IDI]
